# A road damage and life-cycle greenhouse gas comparison of trucking and pipeline water delivery systems for hydraulically fractured oil and gas field development in Colorado

**DOI:** 10.1371/journal.pone.0180587

**Published:** 2017-07-07

**Authors:** Ray C. Duthu, Thomas H. Bradley

**Affiliations:** Department of Mechanical Engineering, Colorado State University, Fort Collins, Colorado, United States of America; Chongqing University, CHINA

## Abstract

The process of hydraulic fracturing for recovery of oil and natural gas uses large amounts of fresh water and produces a comparable amount of wastewater, much of which is typically transported by truck. Truck transport of water is an expensive and energy-intensive process with significant external costs including roads damages, and pollution. The integrated development plan (IDP) is the industry nomenclature for an integrated oil and gas infrastructure system incorporating pipeline-based transport of water and wastewater, centralized water treatment, and high rates of wastewater recycling. These IDP have been proposed as an alternative to truck transport systems so as to mitigate many of the economic and environmental problems associated with natural gas production, but the economic and environmental performance of these systems have not been analyzed to date. This study presents a quantification of lifecycle greenhouse gas (GHG) emissions and road damages of a generic oil and gas field, and of an oil and gas development sited in the Denver-Julesburg basin in the northern Colorado region of the US. Results demonstrate that a reduction in economic and environmental externalities can be derived from the development of these IDP-based pipeline water transportation systems. IDPs have marginal utility in reducing GHG emissions and road damage when they are used to replace in-field water transport, but can reduce GHG emissions and road damage by factors of as much as 6 and 7 respectively, when used to replace fresh water transport and waste-disposal routes for exemplar Northern Colorado oil and gas fields.

## Introduction

Hydraulic fracturing is a key energy technology that has increased the U.S. production of oil and natural gas (O&NG) [1]. As O&NG development has increased in scope, public concern about the potential social, environmental, and economic costs of hydraulic fracturing has increased as well. The loss of this social license to operate can lead to direct and organized public opposition to O&NG development [2]. Much of these costs are attributable to impacts of truck transport of fresh water and wastewater within and outside of the field. To reduce the economic, environmental, and social costs of O&NG, developers have constructed integrated development plans (IDPs) that include pipeline-based fluid transport systems [3].

IDPs, as defined in this study, include a central wastewater processing facility (CPF) connected by pipe to each well (or pad) in an oil and gas field. The CPF treats and recycles the produced water into new fracking fluid, thereby reducing the fresh water requirements for new wells in the field. The CPF is connected by pipeline to a freshwater source as well as a disposal site for untreatable wastewater.

Many of the public’s core concerns with hydraulic fracturing relate to the transport of the wastewater byproduct known as produced water [3] (also referred to in the initial phase as flowback water). The typical business practice for handling freshwater and the wastewater byproduct of O&NG production is to truck these materials to and from the wellsite. The corresponding traffic, decreased road safety, dust, noise, spillage, air quality, habitat degradation, and road damage are cited among the public’s chief concerns with shale gas extraction through hydraulic fracturing [4][5][6]. These externalities have legal and economic consequences for the O&NG well operators [7][8][9] and have often jeopardized the ability of the O&NG to supply their products. Road damage and emissions related to these trucking activities is frequently featured in hydraulic fracturing life cycle analyses (LCAs) and related studies [5],[10],[11],[12],[13],[14]. Some of these studies anticipate a reduction of future costs and damages based on energy companies adopting IDP-based water transport and recycling infrastructure [15],[16], but a lifecycle quantification of these reductions has not been performed, to date.

Previous research into the economic and environmental costs of hydraulic fracturing infrastructure has concentrated on its impacts in regions of the US, other than Colorado. These studies have been demonstrated to have limited utility in describing these costs in Colorado, as they are not directly applicable to the biological and geological features of Colorado or to its local infrastructure and geography [17],[18],[19],[20],[21]. The Colorado Oil and Gas Conservation Commission has encouraged development of IDP technologies and methodologies with systems of pipelines and water management systems already in place in the Piceance Basin [22]. The costs and benefits of pipelines in the O&NG industry is an especially important issue for the state of Colorado in that legislation pertaining to the right of way status for pipeline companies and IDPs is under consideration [23].

These challenges prompt the need to compare pipeline and trucking transport systems across several metrics of economic and environmental performance so as to guide the priorities for development of O&NG fluids management infrastructure in Colorado. This study develops a life cycle assessment of these competing fluid transport systems in a generic field, so as to compare the emissions and road damage costs of construction, installation, operation, and road damage over a twenty year lifetime. Sensitivity cases are used to demonstrate the robustness of the comparison by considering a range of levels of field development and proximity to the surrounding source locations (fresh water and injection wells). Finally, the model is applied to an exemplar O&NG field in the Denver-Julesburg (DJ) Basin of Northern Colorado. The results of this study will provide information to O&NG well operators, policy makers and subsequent LCA studies associated with various design options for the transport of fresh, produced and wastewaters.

## Methods

In order to quantify these differences between IDP pipeline and truck systems, this paper proposes a case study of a set of O&NG fields in the Denver-Julesburg basin of Northern Colorado.

### Data gathering and processing

Publically available analyses are used to derive the required fracking fluid volume per horizontal well in the northern Colorado region [20]. The produced water output for each well as a function of time is modelled for 20 years based on operator-derived data specific to the region [21]. Other aspects of the model are derived from an understanding of the operation and equipment used in field in Denver-Julesburg basin in the northern Colorado region of the US.

### Functional units and scope

The functional units for this study is 1 O&NG well, over its lifetime of 20 years. This functional unit is relevant for studies of environmental and public costs because it allows for direct comparison to previous studies, and it provides input to policy and taxation processes which are assessed on per well basis. All results are presented using metrics of 100Y GWP CO_2_ equivalent units and in 2015 US dollars.

The system boundary for this study includes the direct consumption of energy, fuels and materials used for transportation of O&NG water and waste water. Equipment embedded emissions (for vehicle and pipeline manufacture) and the emissions associated with road repair and re-construction are included in the scope of this study. Because this work assumes that the rural road network of northern Colorado is already developed, we exclude consideration of the emissions associated with road structures, furniture, culverts and earthworks, as might be appropriate for consideration of green-field road construction or road upgrading. Because this study seeks to perform comprehensive analysis of only the water transportation impacts of O&NG production, we do not include emissions or costs associated with O&NG exploration, drilling, fracking, production, fuel use, or end of life.

The case study incorporates a five-year construction period in which all of the wells in field are constructed, and a total 20 year lifetime for the fields. The construction period is organized such that the wells are drilled and fractured at a constant rate—Fields with lower levels of development (fewer total wells) will be drilled at the same rate and thus the field will be fully drilled and fractured in less than the initial five-year period [17].

#### O&NG field model

**Fig 1:**
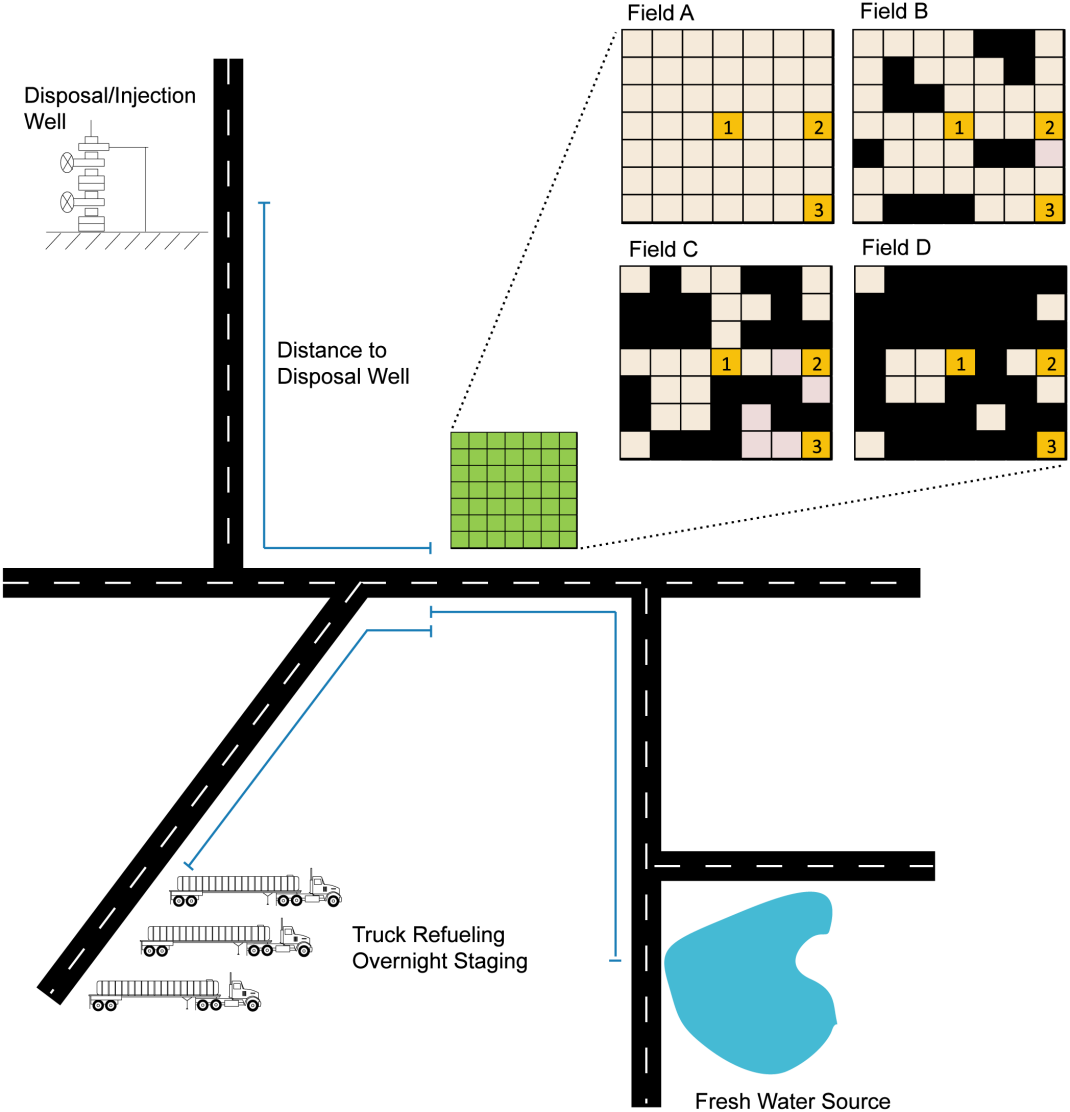
Layout for varying levels of field development (A: 48 sq. miles, B: 36 sq. miles, C: 24 sq. miles, D: 12 sq. miles) and central processing facility locations (1, 2 and 3) and the surrounding field inputs and outputs (Fresh Water, Injection Well and Truck Staging).

An illustration of the generic oil and gas fields under consideration are presented in Fig 1. Each of the 12 variations of the field contains 49 one square mile nodes arranged in seven rows by seven columns, analogous to the township and range lines that define roadways and property lines in rural eastern Colorado. Each node contains two well pads each with eight horizontal wells (for a total of sixteen wells per node). The fields vary as to their level of development (A: 48 nodes per field, B: 36 nodes per field, C: 24 nodes per field, D: 12 nodes per field) and by the location of the central processing facility for water collection and recycling (case 1: centralized, case 2: central edge, case 3: corner). This leads to 12 different field combinations (A1, A2, A3, B1, B2, B3, etc.) for use in this study. All of the roads and pipes within the fields are assumed to travel only in cardinal directions (north, south, east and west, as is characteristic of Northern Colorado O&NG developments). Pipe diameters are assumed to decrease from the centrally-located CPF to the wells as the pipelines split into branch lines, for the purpose of reducing the length of pipe required and thereby reducing capital and installation costs.

Trucking and pipeline costs are strong functions of the proximity of the field to its inputs (such as a fresh water source) and outputs (such as an injection well). The distance to the fresh water source, the produced water injection site and the heavy truck overnight staging/storage and refueling are all independent variables in this case study and are varied through a range of 0–90 miles.

The CPF will recycle the produced water into new fracking fluid in both the trucking and the pipeline models. The fraction of produced water that is recycled into fracking fluid is varied as an independent variable within the range of 0%, 30%, 60% and 100% of the produced water volume. The remaining produced water is trucked or piped to the injection well for disposal.

### Truck transport and emissions model

#### Transport

The truck transport system creates social costs related to increased traffic and road damage, and environmental costs from emissions released during the construction and operation of the trucks and during repair of damaged roads. All of these costs are a function of the distance travelled by the transport trucks that service the field over its 20 year lifetime. Each of the trucks in this model has a transport capacity of 150 barrels (bbl.) of water. Each well requires trucks to deliver fresh water to the CPF, transport the fracking fluid (which may contain some recycled produced water) from the CPF to the well, transport the produced water from the well back to the CPF for recycling, and to deliver the wastewater (un-recycled produced water) from the CPF to a disposal site. Additionally, at the end of each workday, the truck operators will drive the trucks to an overnight staging and refueling site as they return home.

#### Road damage

For this study, road damages and the associated road damage costs are quantified through an Equivalent Single Axle Load (ESAL) analysis developed by the American Association of State Highway and Transportation Officials (AASHTO) [24], [25], [26]. Each water truck has an associated ESAL on asphalt roads while empty (ESAL = 1.363) and filled (ESAL = 2.6) and each of the roads within and surrounding the field has an ESAL design lifetime. Therefore, each truck trip-mile incrementally damages the local road system and reduces each road’s remaining useful lifetime. The majority of roads in Northern Colorado are asphalt [27], so all the roads in the model are assumed to be asphalt. Each of the trucks travels along a generic path represented as including all types of roads (principal arterial, minor arterial, collectors and local roads) based on the average utilization of each of these types of roads in the US [28]. Damage and road repair emissions are allocated to each road type based on their utilization.

In this study, road damage is also quantified using road damage costs, the cost in 2015 USD to repair the road damage caused by the passage of the truck. For this study, we use asphalt road repair costs that vary between $307,200 per lane-mile for principal arterial roads, and $168,000 per lane-mile for local roads. Road damage costs are distributed among the road types based on their assumed utilization. Bridges, which are infrequent in Northern Colorado, but are significantly more costly to repair, are not included in this analysis. The average speed of the trucks on each of these types of roads and the distances traveled influences the number of transport trips that each truck can do per workday.

#### Emissions

Truck transport creates three primary sources of emissions: tailpipe criteria and GHG emissions, truck embedded emissions (associated with truck manufacture), and road repair and construction emissions. Tailpipe or operation emissions is the largest of these three and is released as each truck burns fuel to transport itself and the water from site to site and also while the truck idles during work events (during the period in which the operator fills or empties the storage tank). A 60,000–80,000 lb. (27–36 tonne) truck operates at 5.9 mpg (40 L/100km) while traveling and 0.786 gal/hr. (3.0L/hr.) while idling [29]. Each 1 gallon (3.7L) of diesel emits 24.18 lbs. (10.97 kg) of GHG as it is combusted [30].

Each heavy truck used to transport for the oil and gas field has an average lifetime defined in terms of miles traveled. The travelled distance required to service the all of the wells in the various fields of this study is so great that the embedded emissions related to the construction of the number of trucks required to service the field must also be considered. The construction of each of the trucks in this model releases 116,078 lbs. (52,652 kg) of GHG emissions and each truck has a lifetime of 750,000 miles (1,207,008 km) [31].

### Pipe network and emissions model

The costs and emissions of the pipe network system are calculated with identical boundaries to the truck transport model.

#### Pipeline network

The pipeline system defined in this model has several parts, representing the function and form of real-world in-field water transport systems. A single pump at a fresh water source transports fresh water to the CPF. The CPF has a central pump system capable of delivering the fresh water through in-field pipelines to each node. Each node has a single pump that returns produced water from the pad/wells back to the CPF. Finally, a single pump transports the un-recycled wastewater from the CPF to a disposal site. The “in-field” pipes all run by cardinal direction and are connected and combined at each node to minimize the total length of pipes in the system. That is, if the nodes in field A1 are numbered from left to right and then down by column, a pump in node 1 in the top left corner transports water through nodes 2, 3, 4, 11, and 18 to the CPF at node 25 (see S1 File for more details). The required flowrates are defined such that each node will be able to deliver all of its 16 wells’ produced water output during a time window each day that does not interfere or restrict the other nodes in its branch-line.

The pipeline system creates emissions from four sources: 1) the pumps’ operational emissions created while driving the water to/from the wells and the CPF and while delivering fresh water to and removing wastewater from the CPF, 2) the installation emissions related to trenching and constructing the pipeline network, 3) the embedded emissions related to the construction of the pumps and pipes for the system, and 4) the emissions associated with road damage created by the pipe installation. The inputs to these calculations are presented in Table 1.

**Table 1:** Summary of the material, energy and scenario inputs.

**Embedded Emissions**	**Value**	**Units**	**Notes**
FlexSteel Onshore 2in Diameter Piping	12,638	kgCO2eq/km	54.4% Steel, 48.6%HDPE
FlexSteel Onshore 6in Diameter Piping	59,228	kgCO2eq/km	59.4% Steel, 40.6%HDPE
FlexSteel Onshore 8in Diameter Piping	107,027	kgCO2eq/km	60.8% Steel, 39.2%HDPE
Water Pumps	3.22	kgCO2eq/kg	36.1% Steel, 36.1% Cast Aluminum, 27.8% Copper
Trucks	52,762	kgCO2eq/truck	Ref. [31]
**Pipeline/Road Construction Emissions**	**Value**	**Units**	**Notes**
Pipeline Installation	18,609	kgCO2eq/km	Models a trenched and backfilled pipe installation
Road Construction	272.0	tCO2eq/km/lane	Weighted by road type and utilization
Loaded Truck Road Damage Costs	$0.78	2015 USD/km	Weighted by road type and utilization
Empty Truck Road Damage Costs	$0.41	2015 USD/km	Weighted by road type and utilization
**In-Use Emissions**	**Value**	**Units**	**Notes**
Truck Driving	1.16	kgCO2eq/km	Ref. [29]
Truck Idling	8.62	kgCO2eq/hr.	Ref. [30]
Pumping Electricity	621.8	kgCO2eq/MWhr	Colorado generation mix
**Well Characteristics**	**Value**	**Units**	**Notes**
Drilling Water Required	2769	bbls/well	Representative of DJ Basin, per well
Fracking Water Required	66,476	bbls/well	Representative of DJ Basin, per well
Produced Water Volume	44,191	bbls/well	Representative of DJ Basin, per well

#### Operation emissions

The emissions of operation of the pipeline are primarily derived from the electricity used to pump fresh and wastewater within the pipe network [32]. The total energy consumed by each pump within the system can be estimated as the product of the required mass flow rate (*Q*), the liquid density (*γ*), the pump efficiency (*η*), the output pressure (*P*) and utilization time of each pump in the system (*h*, in hours).

$$W\left[kWh\right]=\frac{P*Q*\gamma }{h}*h$$

The output pressure of the pump must equal the sum of required pressure at the outlet of the pipe, differential height gains/losses and the frictional pressure losses the pipe system (Equation 3). The field in this model is on flat ground and the differential height gains/losses are assumed to be zero. The pressure losses in the pipe system are estimated using the Hazen-Williams equation (Equation 4).

$$P\;=\;P_{outlet}\;+\;\frac{4.52*Q^{1.85}}{C^{1.85}*d^{4.87}}$$

The pipes in this system are based on the FlexSteel^™^ composite pipe product [33] with a Hazen-Williams friction coefficient of *C* = 150. The fresh water, fracking fluid, produced water and wastewater flows are all assumed to have the fluid properties, such as specific gravity (*γ*), of water. The pipe diameter, *d*, varies for each part of the system and was optimized from the options in the FlexSteel^™^ catalog for the minimal combination of pump energy and embedded pipe emissions. All of the pumps in the system have an operating efficiency (*η*) of 0.75, and all parts of the pipeline system are designed for 20 psi (138kPa) static pressure at the outlets.

#### Other emissions

The installation emissions for the pipeline system were estimated based on guidance from the manufacturer and include the operation of a backhoe, excavator, crew truck, drill rig and roller [32][33][34]. To transport both fresh and wastewater between the CPF and the wells, two pipelines are connected to each well, and each pipeline is installed in a single trench. One pipeline and one trench is constructed to connect the CPF to the fresh water source, and the CPF to the wastewater disposal well.

The embedded emissions for the pipeline system the sum of the emissions created during the fabrication of all of the pipes and pumps in the system. The pipe composition (ratios of steel and high-density polyethylene (HDPE)), and mass of the pipe construction materials were estimated from the product specifications [33]. The pump embedded emissions are scaled with pump power [35], and are modeled as having the same emissions per unit mass as electric motors as modeled in the GREET database [30].

Emissions associated with road repair for the installation of the pipeline is calculated using the same method as described in the section on trucking, with a different set of ESALs for trucks that handle the equipment delivery (0.621) and maintenance and operation (0.143).

## Results and discussion

The results of this study are presented in two forms. First, we compare trucking-based water transport and IDP-based water transport for the baseline and sensitivity cases for the generic field model. Then, we compare trucking-based water transport and IDP-based water transport for a field geology, geography, and development plan derived from a case study in Northern Colorado.

### Comparison of truck transport and IDP-based pipeline transport in generic O&NG field

The IDP pipeline systems are shown to reduce both GHG emissions and social costs borne by the local residents in the form of road damages. These benefits are robust across all of the different field types, proximities to water sources and disposal sites, and percentages of waste water that is recycled at the CPF facility.

#### Lifecycle emissions comparison

**Fig 2:**
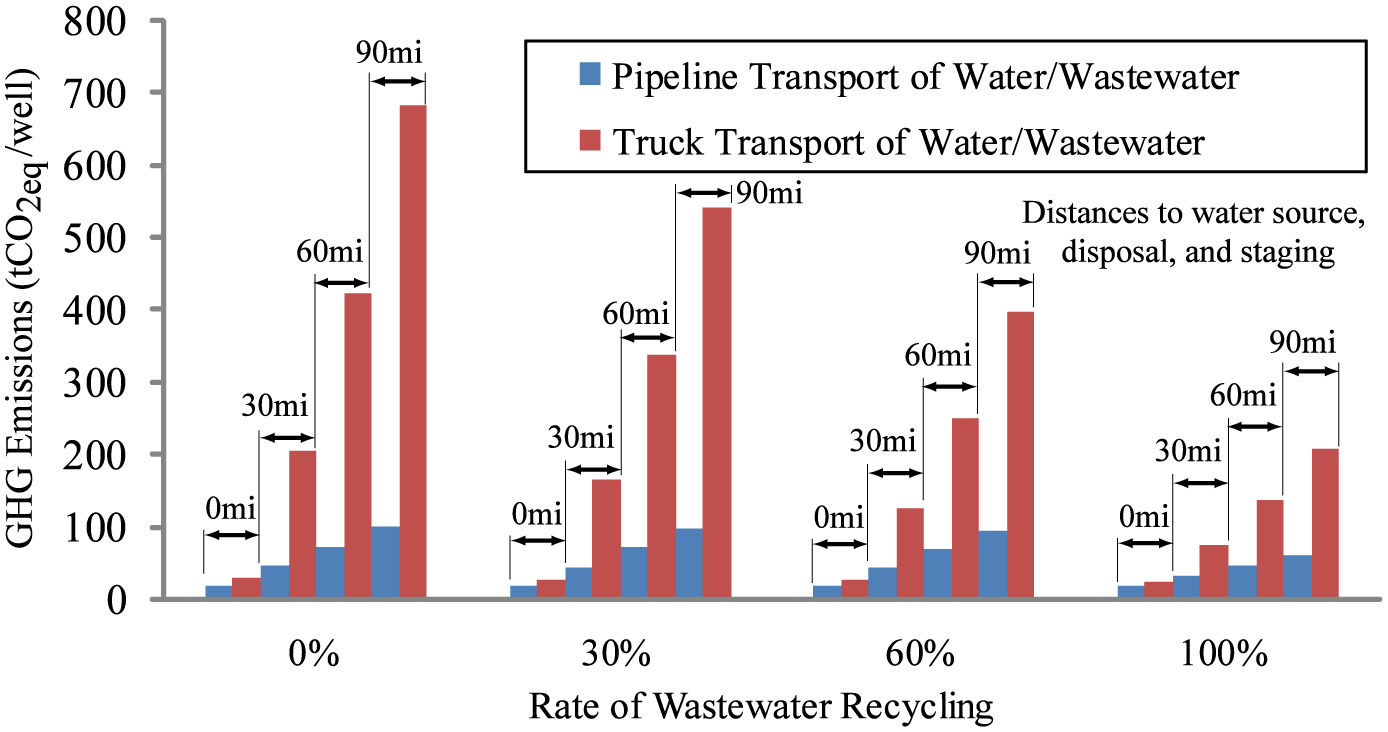
Comparison of pipeline and truck water/wastewater transport methods using the metric of lifetime GHG emissions per well for a range of field proximities to water/wastewater inputs/outputs (60 mi = 97km).

The lifecycle emissions per well for transporting water inputs and outputs to and from the field for a small subset of the cases considered is shown in Fig 2. For each case, the distances to the fresh water site, the disposal site, and the truck refueling and staging location are all assumed equal and are a small subset of the possible combinations (See S2 File for results for more cases). In each case considered for this study, the GHG emissions of truck transport of water/wastewater is significantly higher than the GHG emissions associated with pipeline transport. These conclusions are robust to the breadth of field configurations considered in this study. These results also demonstrate that increased levels of water recycling have the effect of reducing GHG emissions for both methods of water/wastewater transport. Increased rates of wastewater recycling is particularly effective at reducing the GHG emissions of water transport when trucks are used as the means for water/wastewater transportation.

**Fig 3:**
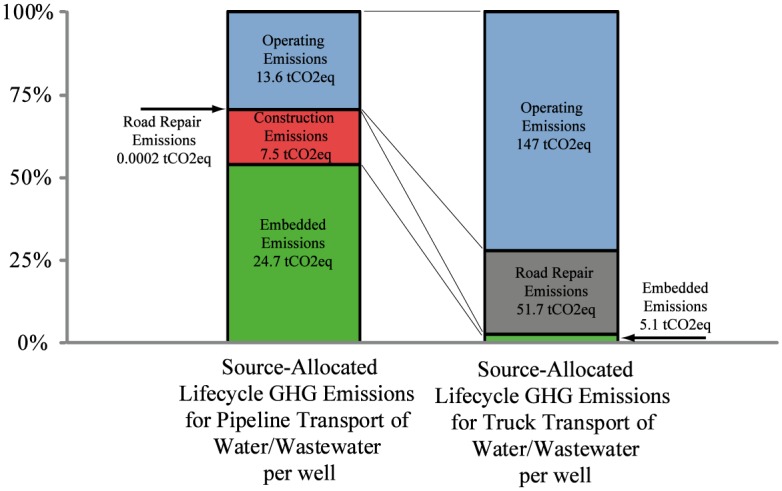
Makeup of lifetime emission per well for baseline field (Field type A1, 30 miles from fresh water, 30 miles from disposal, 30 miles from overnight staging and refueling, and 0% recycling).

Fig 3 illustrates the contributions of each emissions source to the lifecycle GHG emissions associated with piping and trucking systems for a baseline field configuration where we model a near-term feasible water recycling rate of 0%, with the field is located 30 miles from fresh water sources, disposal locations, and truck overnight parking. For the trucking transportation system, the primary source of emissions from the operating (tailpipe) emissions of each truck as it transports the water (147 tCO2eq per well). However, the emissions from asphalt repaving, resurfacing and reconstruction due to road damage are also significant contributors to the truck network lifecycle GHG emissions (51.7 tCO2eq per well). For the pipeline transportation case, GHG emissions is dominated by emissions associated with electrically driven water pumping (operation), emissions generated by the construction of the pipeline, and emissions associated with the pipeline manufacture. Road damage emissions associated with construction and maintenance of the pipeline are negligible in this study. It is notable that the embedded energy of the construction of the pipeline network rather than the operations of the pumps is the largest source of emissions for the system. Hydraulically fractured wells return more produced water in the first few months after completion than over the rest of the lifetime of the well. The utilization rate of the pumps and pipes in the modeled field decreases with time as the flow rate of produced water from the field decreases. In the final 15 years of the project, the water transported by the pipeline system is a small fraction of the system’s capacity, presenting the opportunity to optimize the pipeline system as a function of time. No such re-optimization of the pipeline system is modeled in this simulation.

The results of the lifecycle emissions comparison demonstrate that pipeline-based water/wastewater transportation systems can realize significant environmental benefits when compared to conventional truck transportation systems.

#### Road damage costs

The road damage comparison presented in Table 2 demonstrates that trucking systems create much road damage costs than do IDP pipeline systems. Under our baseline scenario, the road damage (reconstruction) costs of the pipeline network ($276 per well) can be compared to the road damage costs of the trucking transport system ($63,250 per well). This study demonstrates that a majority of the road damage is incurred during the trip to and from the remote well site, and that the well density, CPF location, and in-field travel paths has a negligible effect on the road damage costs. Because recycling of wastewater into fracking fluid reduces the number of trips to freshwater uptake and wastewater disposal sites, increased recycling rates reduce road damage costs.

**Table 2:** Summary of road damage costs.

Summary of Road Damage Costs	Pipeline System	Trucking System	Units
**Baseline (Field Type A, 30mi to water sources, disposal, and staging, 0% recycling)**	**$ 276**	**$ 63,250**	**USD/well**
Field Type A, central CPF, 30mi to water sources, disposal, and staging, 30% recycling	$ 276	$ 50,850	USD/well
Field Type A, central CPF, 30mi to water sources, disposal, and staging, 60% recycling	$ 276	$ 38,380	USD/well
Field Type A, central CPF, 30mi to water sources, disposal, and staging, 100% recycling	$ 200	$ 21,790	USD/well
Field Type B, central CPF, 30mi to water sources, disposal, and staging, 0% recycling	$ 328	$ 63,210	USD/well
Field Type C, central CPF, 30mi to water sources, disposal, and staging, 0% recycling	$ 451	$ 63,130	USD/well

These costs can be compared with those of previous studies, such as the Boulder County road damage study which found that each well drilled in Boulder County would create $30,600 in roadway costs for roads in Boulder County alone [26]. This number was later revised down to $20,600 by the Boulder County Board of Commissioners after a review of local truck travel information. The cost in this Boulder study represents the damages to roads only within the relatively small (751 square miles, approximately 27 miles in width and height), and urbanized Boulder County, and any damages outside the county were excluded. The case study presented here considers all of the road damage for all roads in all counties and also includes consideration of the longer travel distances that are more relevant to rural Colorado, including Weld County.

**Fig 4:**
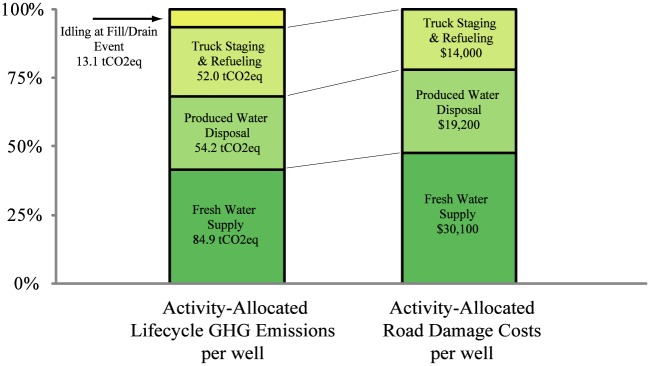
Makeup of lifetime truck transport emissions and road damage costs based on truck activity, per well for baseline field (Field type A1, 30 miles (48 km) from fresh water, 30 miles (48 km) from disposal, 30 miles (48 km) from overnight staging and refueling, and 0% recycling).

Fig 4 breaks down the emissions and road damage costs associated with each type of truck transport action for the baseline field configuration (Field A1, 30 miles (48 km) from each of the field inputs and outputs and 0% recycling). Over 20 years, each well returns much of the fresh-water-derived fracking fluid as produced water, but fresh water supply remains the largest transport requirement. The Fill / Drain events relate to the amount of time each truck spends idling while either waiting in line to deliver fracking fluid to a well for completion or while the operator fills or drains the truck tank with water.

This generic case study was built to represent the set of field configurations that could exist in Northern Colorado. The resulting comparison of truck-based water/wastewater transport system to a pipeline-based IDP system demonstrate that the pipeline based system exhibits lower road damage costs, and lower lifecycle GHG emissions. These results are robust to the variability in geography and recycling rates that were considered within this simulation.

### Application to East Pony field in Weld, County, Colorado

To complete this comparison of pipeline-based and trucking-based water transport emissions and social costs, we present the comparison applied to an actual oil and gas development. The East Pony O&NG field is located in the Denver-Julesburg basin in northeastern Colorado (approximate location is at 40°44'34.8"N 103°57'00.0"W). This field is considerably larger and has more wells than any of the types presented in this case study, though it is most similar to the A1 field type. The average distance to the nearest four water sources of suitable capacity is 31.1 miles (50 km) (“Rohn pond”, “Everitt well”, “North Timmerman pond” and the “Hwy 52 well”). The average distance to the nearest two injection wells is 42.8 miles (68 km) (“High Sierra C7” in Cornish, Colorado and “C8” in Grover, Colorado). The distance to Lucerne, Colorado (a common location for truck operators to refuel and store vehicles overnight) is 65.4 miles (105 km) from the East Pony field.

**Fig 5:**
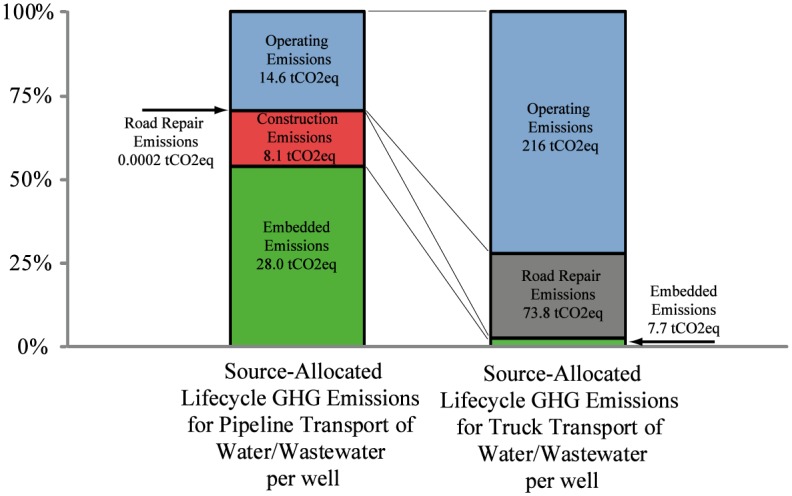
Makeup of lifetime emission per well for East Pony, Weld County, Colorado (31.1 miles (50 km)from fresh water, 42.8 miles (68 km) from disposal, 65.4 miles (105 km) from overnight staging and refueling, and 0% recycling).

**Fig 6:**
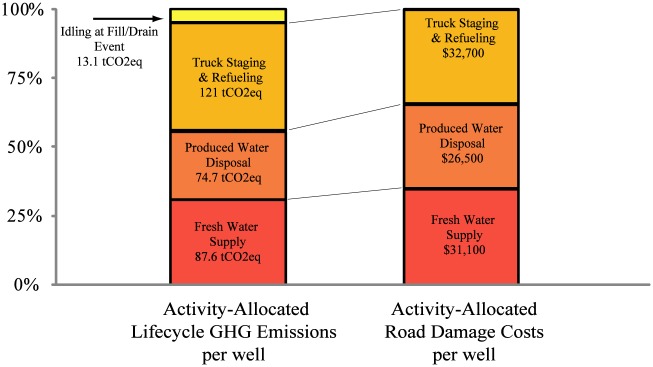
Makeup of lifetime truck transport emissions and road damage costs based on truck activity, per well for East Pony, Weld County, Colorado (31.1 miles (50 km) from fresh water, 42.8 miles (68 km) from disposal, 65.4 miles (105 km) from overnight staging and refueling, and 0% recycling).

Fig 5 compares the emissions for pipeline and truck water transport for the East Pony field using the A1 case as the production plan. These results demonstrate again, that the lifecycle emissions associated with truck transport of fluids within and to/from the field (297 tCO2eq GHGs per well) is much higher than the emissions associated with fluid transport using pipeline networks (50.6 tCO2eq GHGs per well). Both road repair and vehicle emissions contribute significantly to the GHG emissions of truck transport. As shown in Fig 6, the road damage costs associated with truck transport over the life of the well are approximately $90,200 per well for the East Pony O&NG development. That this valuation of road damage is higher than has been published in analyses of Marcellus formation is primarily due to the remoteness of the site, and the large distances travelled between water sources, wastewater disposal sites, and truck refueling and staging facilities [12].

## Conclusions

Hydraulic fracturing is a key component of the US’ future energy economy, but it creates significant social and environmental costs for local communities. Many of these costs could be mitigated by transitioning water transport from trucking systems to an IDP including pipeline water transport systems. Pipeline systems are generally considered safer and historically spill less hazardous material than trucks [36], but the location of pipeline spills may increase the severity of any spill [37], so cooperation of utilities and local governing agencies while establishing the pipeline routes should be a priority. Through a lifecycle GHG emissions comparison of these technologies, pipeline systems are demonstrated to uniformly emit less GHGs than trucking systems and dramatically reduce damages and costs to the local road infrastructure, especially for long range bulk transport of water to and from freshwater sources or disposal wells. O&NG development operations already have shown interest in developing and constructing IDPs as they are asserted to also reduce the operational costs of water transport. This research offers an estimate of the social and environmental value of these IDP systems. Future work can explicitly calculate the operational economic benefits and costs of switching to IDPs for specific oil and gas fields with site specific pipeline optimization methods.

## Supporting information

S1 File Background, risk analysis, and supplementary results document. (DOCX)

S2 File Data and analysis worksheet. (XLSM)
