# Supplementary material for: A road damage and life-cycle greenhouse gas comparison of trucking and pipeline water delivery systems for hydraulically fractured oil and gas field development in Colorado
Source: PLoS One. 2017 Jul 7;12(7):e0180587. doi: 10.1371/journal.pone.0180587 (PMC5501573; doi:10.1371/journal.pone.0180587)
Supplement: S1 File — (DOCX) [file pone.0180587.s001.docx]

Supplementary Information for: A Road Damage and Life-Cycle GHG Comparison of Trucking and Pipeline Water Delivery Systems for Hydraulically Fractured Oil and Gas Field Development in Colorado

Ray C. Duthu,

Department of Mechanical Engineering, Colorado State University, Fort Collins, CO 80523-1374

[Ray.Duthu@gmail.com](mailto:Ray.Duthu@gmail.com)

Thomas H. Bradley^[[1]](#footnote-1)^

Systems Engineering, Colorado State University, Fort Collins, CO 80523-1301

[Thomas.Bradley@colostate.edu](mailto:Thomas.Bradley@colostate.edu)

This research was funded through grants provided by the Research Partnership to Secure Energy for America (RPSEA).

[Supplementary Information for: A Road Damage and Life-Cycle GHG Comparison of Trucking and Pipeline Water Delivery Systems for Hydraulically Fractured Oil and Gas Field Development in Colorado 1](#_Toc471293651)

[Hydraulic Fracturing Background 2](#_Toc471293652)

[Risk Analysis of Pipeline vs. Trucking Transportation Systems 3](#_Toc471293653)

[Supplementary Results 4](#_Toc471293654)

[References 6](#_Toc471293655)

# Hydraulic Fracturing Background

Anderson and Theodori’s interviews into the reactions to hydraulic fracturing in the Barnett shale demonstrate that the core public concerns with hydraulic fracturing relate to the transport of the wastewater byproduct known as produced water (also referred to in the initial phase as flowback water). Residents in the Barnett study cited increased traffic, decreased road safety, and road damage [1] as among their chief concerns with hydraulic fracturing. Other studies have shown that public roads in counties that do not receive any benefits from gas drilling could also be adversely affected [2]. Randall found that dust, noise and road damage are on the top of citizen concerns in areas where shale gas is extracted [3]. Cooley and Donnely also note concerns over wastewater, spillage, and air quality based on interviews with various state and federal representatives [4]. The local costs associated with hydraulic fracturing are at such a level that Theodori and Anderson have shown that while local residents of the Barnett shale describe positive attitudes about the potential benefits of hydraulic fracturing at the onset of new drilling/fracturing, public opinion in previously explored regions of the play has significantly decreased towards a plurality of disapproval over a span of ten years. For instance, at one point group of citizens in Texas attempted to block the construction of an injection well through the court system with the argument that the increased truck traffic and road damage would not be in the public interest [5]. Greater public education on the potential costs and benefits of oil and gas development may improve public opinion at the onset of development [6], but if the current hydraulic fracturing practices create such a negative impact on community opinion in once-receptive regions, this will likely only exacerbate public opposition in regions already hostile to new or expanded oil and gas operations.

In 2009 Clark and Veil noted that trucking and injecting wastewater was the typical business practice for handling this wastewater byproduct, but noted that there was definite potential for an improvement of transport and for a beneficial reuse [7]. Road damage and emissions related to trucking fresh and produced water is frequently featured predominately in hydraulic fracturing LCAs and related studies [2],[8],[9],[10],[11],[12]. Some of these life cycle analyses anticipate a significant reduction of future costs and damages based on energy companies adopting more IDP friendly plans – piping water rather than trucking and beginning to clean and recycle the wastewater byproduct [13],[14]. Pipeline systems and recycling could offer direct economic benefit to the field developers as well – truck transportation is the most expensive aspect of water handling [12].

The primary challenges related to the successful implementation of IDPs for oil and gas fields are not technical. Various industries and local municipalities have been transporting, filtering and treating water for decades, and there are already numerous technological possibilities for transferring the state of the art into transporting and recycling produced water [15]. Tailoring these processes into an integrated water management plan for oil and gas fields requires a detailed understanding of the water use and characterization of the wastewater (produced water) in these fields [16]. Additionally, quantifying the potential economic, social and environmental costs and benefits associated with integrated development plans requires a model that can estimate both the impacts of these integrated development plans and the costs associated with a more traditional and less integrated truck delivery of water supplies to and from the fields. In support IDP planning in the Denver-Julesburg basin, Goodwin, Bai and Carlson have modeled both freshwater requirements as well as the volume and composition of the produced water byproducts associated with hydraulic fracturing in the northern Colorado region [17],[18],[19] with the ultimate goal of modeling the feasibility of integrated development plans (IDPs) and developing a toolset to help energy companies improve their decision making process [16].

In order to expand and continue operations, energy companies must manage not only costs and find new technologies for harvesting natural resources economically; they must also manage public opinion and operate as cleanly as possible with minimal social and environmental impact. IDPs have the potential to improve both the economics and the social license to operate for hydraulic fracturing, but they are difficult to plan, require more significant upfront costs than trucking, and their economic, social, and exact environmental benefits are still unknown. The Colorado Oil and Gas Conservation Commission has previously encouraged development of these technologies and methodologies with pipelines and water management systems already in place in the Piceance Basin [20]. This remains a key issue for the state of Colorado with legislation pertaining to the right of way status for pipeline companies and IDPs under consideration [21].

Based on our understanding of the field, the goal of this research is to compare pipeline and trucking transport systems across several metrics. The current state of the art expressed in the literature in this field is used to quantify and compare the risks of incidents, injuries and spillage for both of these systems. A model of an oil and gas field generic to the northern Colorado Denver-Julesburg basin is developed and then used to compare the local social cost (road damage) and environmental impact (greenhouse gas or GHG emissions) associated with truck transport and pipeline systems. Additionally, variations of this generic field model will quantify the beneficial impact of reducing the fresh and wastewater transport requirements by recycling produced water into new fracking and prove the robustness of the comparison by including a range of levels of field development and proximity to the surrounding source locations (fresh water and injection wells).

## Risk Analysis of Pipeline vs. Trucking Transportation Systems

Increased traffic and accidental spills are major local safety concerns related to the transport of water for hydraulic fracturing [22]. Produced water spills may adversely affect both terrestrial and aquatic environments. The chemical and physical composition of produced water is dependent on the source geology, but typically includes organic constituents and hydrocarbons, metals, salts and other dissolved and suspended solids and the remnants of the injection fluid and proppants (sand/silt). Grubert and Kitasei describe the truck transport of produced water as one of the major pathways to water contamination [23]. Pipeline systems eliminate nearly all of the truck miles associated with water transport, and thus improve road safety, but both systems carry risks of accidental or uncontrolled spills of hazardous materials.

The average fatal crash rate for all vehicle types for the state of Colorado is approximately one fatal crash per 100 million vehicle miles travelled (VMT); but heavy trucks create higher risks. According to the US Dept. of Transportation, the fatal crash rate for single trailer trucks ranges from ~1.5 to ~4.5 fatal crashes per 100 million VMT depending on road type. Depending on location, hydraulically fracturing a single well can create thousands to hundreds of thousands of truck miles associated with water transport alone. Truck transport of the water demands for a field of hundreds or more wells multiplies a significant risk of fatal vehicular accidents. Some measure of increased truck traffic would occur during the construction of a pipeline system, but as shown in the results, it is insignificant compared to the traffic associated with truck-based water transport systems.

In terms of public safety, pipeline systems are generally considered safer than truck transport. Between1994 to 2009, trucks were responsible for several times as many incidents, injuries and fatalities as pipelines per ton-mile of transport of hazardous materials [24],[25]. Figure 0.1, constructed from data combined from the US Dept. of Transportation Bureau of Transportation Statistics [24] and the American Petroleum Institute [25] demonstrates that historically in the transport of crude and refined oil products, trucks have spilled more barrels per barrel-mile of transport than pipelines since 1980. However, the magnitude of spilled material involved in each truck accident is generally lower than the material spilled in a pipeline accident (limited to the size of the carrying capacity of a truck/trailer combination). Also, the hazardous material is generally spilled on top of relatively impermeable and low-impact road surfaces. Individual pipeline spills have the potential to spill a larger quantity of fluid per spill and these spills may occur on much more ecologically vulnerable surfaces and closer to water resources [26].

Pipeline systems could offer a benefit compared to truck transport of reduced spill volume of environmentally damaging produced water as long as the pipeline network is well designed according to best industry practices and is located sufficiently far from ecologically vulnerable areas or fresh water sources. The research presented in this paper assumes that the pipeline systems are located adjacent to the same roads that would be used for truck transport to minimize the increase spill vulnerability and to maintain a generic transportation distance for comparison between the two transport methods. This may not be a realistic expectation for each oil and gas field and coordination with local governing agencies may be required to ensure that pipeline systems are well located such that the damage related to accidental spills, which will inevitably occur, can be adequately managed and contained.


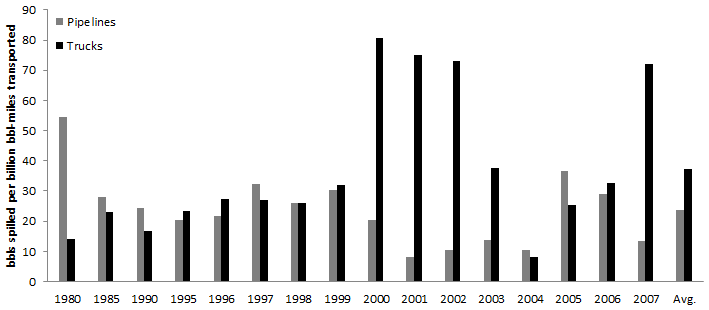


Figure 0.1: Combined Crude Oil and Petroleum Spills during Domestic Transportation

### Supplementary Results

The GHG emissions associated with the piping networks proposed in an IDP compared to the emissions of a traditional trucking system for all the field types in this case study are presented in Figure 0.2. These are the in-field emissions only, they do not include emissions for fresh water transport to the field, wastewater removal to the disposal site, or for refueling and staging the transport trucks. The IDP pipe network shows a robust reduction of GHG emissions within the field in all cases. Trucking systems are adversely affected if the CPF is not optimally located within the center of the field, but are not directly affected by the level of development in the field. The slight decline in truck emissions per well from the A-type fields to the D-type fields is due to a slightly unsymmetrical elimination of nodes within the fields (more nodes that were distant to the CPF than were near were removed in D). Piping system emissions are less affected by CPF location, but are best utilized in densely developed fields.


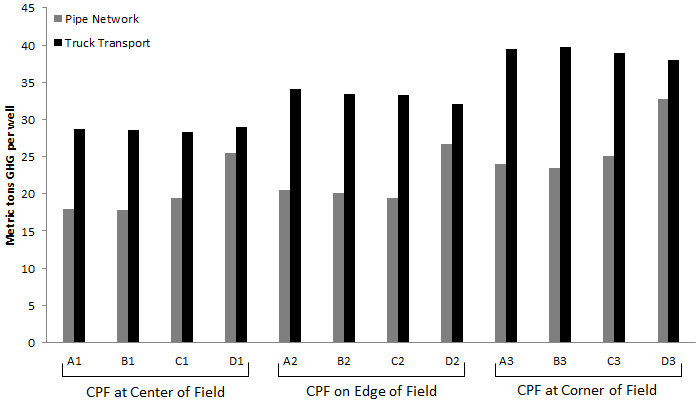


Figure 0.2: Lifetime Emissions per Well Comparison for In-field only Transport

The road damage comparison in Figure 0.3 demonstrates that trucking systems create overwhelmingly larger social costs than IDP pipeline systems.


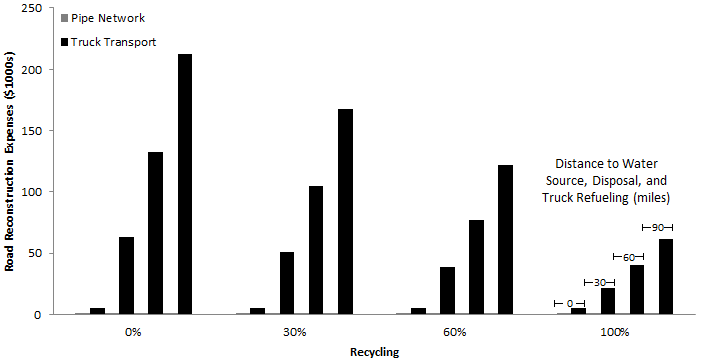


Figure 0.3: Lifetime Road Damage Costs Comparison for a Range of Field Proximities to Inputs/Outputs

References

1. Jolanda Prozzi, Sergey Grebenschikov, Ambarish Banerjee & Jorge Prozzi, Impacts of Energy Developments on the Texas Transportation System Infrastructure, Center for Transportation Research at the University of Texas at Austin (2011).
2. Susan Christopherson & Ned Rightor, How Shale Gas Extraction Affects Drilling Localities: Lessons for Regional and City Policy Makers, Journal of Town & City Management, Vol. 2, No. 4 (2011) at 350.
3. C. J. Randall, Hammer Down: A Guide to Protecting Local Roads Impacted by Shale Gas Drilling, (2010) at http://www.greenchoices.cornell.edu/downloads/development/shale/Protecting_Local_Roads.pdf (last visited: Jan 23, 2015).
4. Heather Cooley & Kristina Donnelly, Hydraulic Fracturing and Water Resources: Separating the Frack from the Fiction, Pacific Institute for Studies in Development, Environment & Security, (2012).
5. R.R. Comm’n of Tex. v. Tex. Citizens for a Safe Future & Clean Water, 336 S.W.3d 619, 622–23, 633 (Tex. 2011) at: http://www.leagle.com/decision/In%20TXCO%2020110311708.xml/RAILROAD%20COM%27N%20v.%20CITIZENS%20FOR%20SAFE%20FUTURE (last visited: June 2, 2015).
6. Hilary Boudet, Christopher Clarke, Dylan Bugden, Edward Maibach, Connie Roser-Renouf & Anthony Leiserowitz, “Fracking” Controversy and Communication: Using National Survey Data to Understand Public Perceptions of Hydraulic Fracturing, Energy Policy, Vol. 65 (2014) at 57-67.
7. C. E. Clark & J. A. Veil, Produced Water Volumes and Management Practices in the United States, Argonne National Laboratory, (2009) doi:10.2172/1007397.
8. Shmuel Abramzon, Constantine Samaras, Aimee Curtright, Aviva Litovitz & Nicholas Burger, Estimating the Consumptive Use Costs of Shale Natural Gas Extraction on Pennsylvania Roadways, Journal of Infrastructure Systems, Vol. 20, No. 3 (2014) at 06014001-1-5.
9. Pam Boschee, Handling Produced Water From Hydraulic Fracturing, Oil and Gas Facilities (2012) at 22-26.
10. Mohan Jiang, W. Michael Griffin, Chris Hendrickson, Paulina Jaramillo, Jeanne VanBriesen & Aranya Venkatesh, Life Cycle Greenhouse Gas Emissions of Marcellus Shale Gas, Environmental Research Letters, Vol. 6 (2011) at 034014 1-9.
11. Aviva Litovitz, Aimee Curtright, Shmuel Abramzon, Nicholas Burger & Constantine Samaras, Estimation of Regional Air-Quality Damages from Marcellus Shale Natural Gas Extraction in Pennsylvania, Environmental Research Letters, Vol. 8 (2013) at 014017 1-8.
12. Daniel J. Stepan, Richard E. Shockey, Bethany A. Kurz, Nicholas S. Kalenze, Robert M. Cowan, Joshua J. Ziman & John A. Harju, Bakken Water Opportunities Assessment – Phase 1, Energy & Environmental Research Center (2010) at: http://www.undeerc.org/bakken/pdfs/FracWaterPhaseIreport.pdf (last visited June 4, 2015).
13. Alexander T. Dale, Vikas Khanna, Radisav D. Vidic & Melissa M. Bilec, Process Based Life-Cycle Assessment of Natural Gas from the Marcellus Shale, Environmental Science and Technology, Vol. 47, No. 10 (2013) at 5459-5466.
14. Trevor Stephenson, Jose Eduardo Valle, & Xavier Riera-Palou, Modeling the Relative GHG Emissions of Conventional and Shale Gas Production, Environmental Science and Technology, Vol. 45, No. 24 (2011) at 10757-10764.
15. Colorado School of Mines, An Integrated Framework for Treatment and Management of Produced Water, RPSEA Project 07122-12 (2009).
16. Stephen Goodwin, Water Management and Reuse Strategies for Unconventional Oil and Gas Fields (2014) (unpublished Ph.D dissertation, Colorado State University) (on file with author).
17. Stephen Goodwin, Ken Carlson, Bing Bai, Luke Rein, Ken Knox & Caleb Douglas, Improved Water Use Estimates for Drilling and Hydraulic Fracturing in Northeastern Colorado, Journal of Water Resources and Protection, Vol. 5, No. 12 (2013) at 1262-1267.
18. Bing Bai, Stephen Goodwin & Ken Carlson, Modeling of Frac Flowback and Produced Water Volume from Wattenberg Oil and Gas Field, Journal of Petroleum Science and Engineering, Vol. 108, (2013) at 383-392.
19. Equations for Flowback, Transition, and Produced Water for the East Pony region, Bing Bai unpublished work (2014).
20. Colorado Division of Water Resources, Colorado Water Conservation Board & Colorado Oil and Gas Conservation Commission, Water Sources and Demand for the Hydraulic Fracturing of Oil and Gas Wells in Colorado from 2010 through 2015, at: https://cogcc.state.co.us/Library/Oil_and_Gas_Water_Sources_Fact_Sheet.pdf (last visited: Feb. 23, 2015).
21. Colorado Senate Bill 14-093 at: http://www.leg.state.co.us/clics/clics2014a/csl.nsf/fsbillcont/E4292BBC297E713687257C360075F8F5?Open&file=093_ren.pdf (last visited Jul. 23, 2014).
22. Hannah J. Wiseman, Risk and Response in Fracturing Policy, University of Colorado Law Review, Vol. 84 (2013) at 729-817.
23. Emily Grubert & Saya Kitasei, How Energy Choices Affect Fresh Water Supplies: A Comparison of U.S. Coal and Natural Gas, Worldwatch Institute (2010) at http://www.worldwatch.org/system/files/pdf/natural_gas_BP2_nov2010.pdf (last visited Jan. 23, 2015).
24. U.S. Department of Transportation Bureau of Transportation Statistics, National Transportation Statistics, at http://www.bts.gov/publications/national_transportation_statistics (last visited: May 28, 2015).
25. Dagmar Schmidt Etkin, Analysis of U.S. Oil Spillage, API Publication 356 (2009).
26. U.S. Department of Transportation Pipeline and Hazardous Materials Safety Administration, Building Safe Communities: Pipeline Risk and its Application to Local Development Decisions (2010) at https://primis.phmsa.dot.gov/comm/publications/PIPA/PIPA-PipelineRiskReport-Final-20101021.pdf (last visited June 2, 2015).
27. Yong Bai, Steven D. Schrock & Thomas E. Mulinazzi, Estimating Highway Pavement Costs Attributed to Truck Traffic, Kansas University Transportation Research Institute (2009) at http://digitalcommons.unl.edu/cgi/viewcontent.cgi?article=1054&context=matcreports (last visited Jan. 20, 2015).
28. American Association of State Highway and Transportation Officials, Guide for Design of Pavement Structures, 4th ed. (1993).
29. Felsburg Holt & Ullevig and BBC Research and Consulting, Boulder County Oil and Gas Roadway Impact Study, Boulder County, FHU Reference No. 12‐109‐01 (2013).
30. Colorado Department of Transportation, 2011 Fact Book, at https://www.codot.gov/library/FactBook/FactBook2011/at_download/file (last visited: May 28, 2015).
31. U.S. Department of Transportation Federal High Administration, Flexibility in Highway Design, at http://www.fhwa.dot.gov/environment/publications/flexibility/ch00.cfm (last visited: May 28, 2015).
32. US Dept. of Transportation, Traffic Safety Facts 2012, at http://www-nrd.nhtsa.dot.gov/Pubs/812032.pdf (last visited June 1, 2015).
33. Linda Gaines, Anant Vyes & John L. Anderson, Estimation of Fuel Use by Idling Commercial Trucks, Center for Transportation Research, Argonne National Laboratory, Paper No. 06-2567 (2006).
34. Argonne National Laboratory, GREET 1 2014, (2014) at: https://greet.es.anl.gov/ (last visited (June 4, 2015).
35. Argonne National Laboratory, GREET 2 2014, (2014) at: https://greet.es.anl.gov/ (last visited (June 4, 2015).
36. The World Bank, Greenhouse Gas Emissions Mitigation in Road Construction and Rehabilitation A Toolkit for Developing Countries (2011) at: https://www.astae.net/sites/astae/files/publication/GHG-Web-final.pdf (last visited: May 28, 2015).
37. Linda Gaines, Frank Stodolsky, Roy Cuenca & James Eberhardt, Life-Cycle Analysis for Heavy Vehicles, Air & Waste Management Association Annual Meeting (1998) at http://www.transportation.anl.gov/pdfs/TA/102.pdf (last visited June 3, 2015).
38. Kalyan R. Piratla, Samuel T. Ariaratnam & Aaron Cohen, Estimation of CO2 Emissions from the Life Cycle of a Potable Water Pipeline Project, Journal of Management in Engineering Vol. 28, No. 1 (2012) at 22-30.
39. FlexSteel, FlexSteel Pipe Specification Sheet, at http://www.flexsteelpipe.com/ (last visited: May 28, 2015).
40. Samuel T. Ariaratnam & Shaik S. Sihabuddin, Comparison of Emitted Emissions between Trenchless Pipe Replacement and Open Cut Utility Construction, Journal of Green Building, Vol. 4, No. 2 (2009) at 126–140.
41. Tuma, Nocchi Pentair Water Electric Pumps Catalogue, at: http://www.tumapumpen.at/upload/medialibrary/16_Katalog.pdf (last visited: May 28, 2015).
42. Boulder County Board of County Commissioners Public Hearing, May 16, 2013, 4:00PM at: http://www.bouldercounty.org/doc/landuse/dc120003staffrec20130516.pdf (last visited May 28, 2015).
43. New York State Department of Environmental Conservation, Supplemental Generic Environmental Impact Statement on the Oil, Gas and Solution Mining Regulatory Program, (2009).
44. Google Maps, 40°44'34.8"N 103°57'00.0"W, Earth Map, https://www.google.com/maps/place/40%C2%B044'34.8%22N+103%C2%B057'00.0%22W/@40.6591391,-104.5861605,114202m/data=!3m1!1e3!4m2!3m1!1s0x0:0x0 (last visited: June 7, 2015).

1. Corresponding author [↑](#footnote-ref-1)
